# Staged Therapy of Polyaneurysmal Disease Including the World's First BeFlared Use in Anaconda FEVAR

**DOI:** 10.1055/a-2591-9692

**Published:** 2025-05-26

**Authors:** Paula R. Keschenau, Sharif Elshafei, Johannes Kalder

**Affiliations:** 1 Department of Adult and Pediatric Cardiovascular Surgery, University Hospital Giessen and Marburg Campus Giessen, Giessen, Germany

**Keywords:** aortic aneurysm, peripheral aneurysm, fenestrated endovascular aortic repair, endovascular aortic repair, bridging stent graft

## Abstract

**Background:**

Abdominal aortic aneurysms frequently coincide with popliteal artery aneurysms. The association with multiple peripheral aneurysms, sometimes called polyaneurysmal disease, is less frequent.

**Case Description:**

An 82-year-old male was diagnosed with polyaneurysmal disease. He was treated by femoral, popliteal, and profundal interposition grafts as well as fenestrated endovascular repair (FEVAR) using an Anaconda (Terumo Aortic, Inchinnan, Scotland) endoprosthesis combined with BeGraft peripheral and BeFlared (BF) bridging stent grafts (Bentley InnoMed, Hechingen, Germany).

**Conclusion:**

Polyaneurysmal disease in an aged patient can be treated successfully by thorough case planning, staging of procedures, and combining different vascular surgical techniques. It demonstrates the smooth implantation of the BeFlared in an Anaconda FEVAR.

## Introduction

The high co-prevalence of popliteal artery aneurysms (PAAs) and abdominal aortic aneurysms (AAA) is well known. 1 The coexistence of AAA and multiple peripheral arterial aneurysms without being based on a connective tissue disorder, sometimes called polyaneurysmal disease, 2 is, however, less common. 3

The present case illustrates how intermittent claudication (IC) can be just “the tip of the iceberg” and how an aged patient can be treated successfully by a staged approach. At the same time, it reports the first implantation of the new BeFlared (BF) stent graft (Bentley InnoMed Hechingen, Germany) as a bridging stent graft in fenestrated endovascular aortic repair (FEVAR) using the Anaconda endoprosthesis (Terumo Aortic, Inchinnan, Scotland) as the main device.

## Case Description

An 82-year-old male patient was referred from his general practitioner for a vascular surgical consultation because of IC in his right calf with a very short walking distance.

**Fig. 1:**
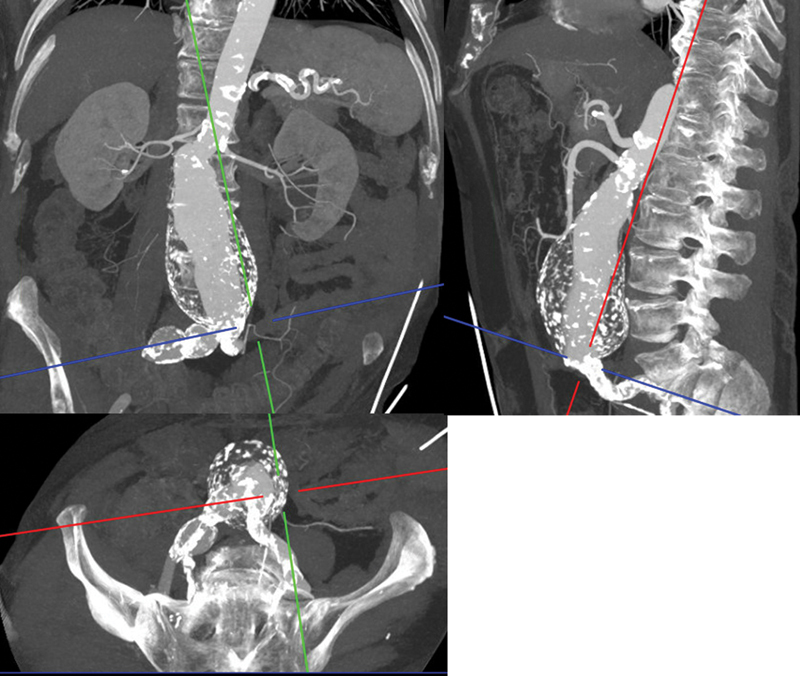
Multiplanar reconstructions of the preoperative CTA scan showing the large juxtarenal abdominal aortic aneurysm (AAA) and right common iliac artery aneurysm with subtotal occlusion of the right internal iliac artery. CTA, computed tomography angiography.

Duplex ultrasound of the femoro-popliteal arteries showed on the right side a common femoral artery (CFA) aneurysm (maximum diameter 26 mm) with a postaneurysm stenosis of the femoral artery bifurcation and an occluded PAA (maximum diameter 20 mm) alongside advanced arteriosclerotic disease of the crural arteries. On the left side, it showed an aneurysm of the deep femoral artery (DFA; maximum diameter 26 mm) and a still perfused PAA (maximum diameter 21 mm) with the presence of thrombus. The patient underwent a computed tomography angiography scan, revealing additionally a large (72 mm) juxtarenal AAA, a right common iliac artery (CIA) aneurysm, and ectatic left CIA (maximum diameter 15 mm; Fig. 1 ).

As for his medical history, he had had coronary artery bypass surgery 24 years ago, suffered from arterial hypertension, hyperlipidemia, had a family disposition for cardiovascular arteriosclerotic disease, as well as a history of prostate cancer with hormone therapy. A genetic disorder favoring arterial aneurysms had never been investigated.

The patient was in very good general condition for his age, and the preoperative cardiological evaluation showed a good cardiac function despite his history. After discussing the therapeutic options and obtaining his informed consent, he was scheduled for a staged repair of the multiple aneurysms: First, the right CFA aneurysm was repaired with an 8-mm Dacron interposition graft and distalization of the femoral artery bifurcation. The operation and the postoperative course were uneventful, and the patient was discharged home after 7 days. He reported an improvement of the IC afterward, so that no revascularization for the occluded right PAA was planned.

To repair the aorto-iliac aneurysms, an endovascular approach by means of FEVAR with simultaneous repair of the left DFA aneurysm was planned, and a custom-made Anaconda endograft (Terumo Aortic, Inchinnan, Scotland) was ordered.

During the manufacturing time, the left-sided PAA was repaired by a popliteo-popliteal venous interposition graft from a dorsal approach. This operation and the postoperative course were also uneventful.

**Fig. 2:**
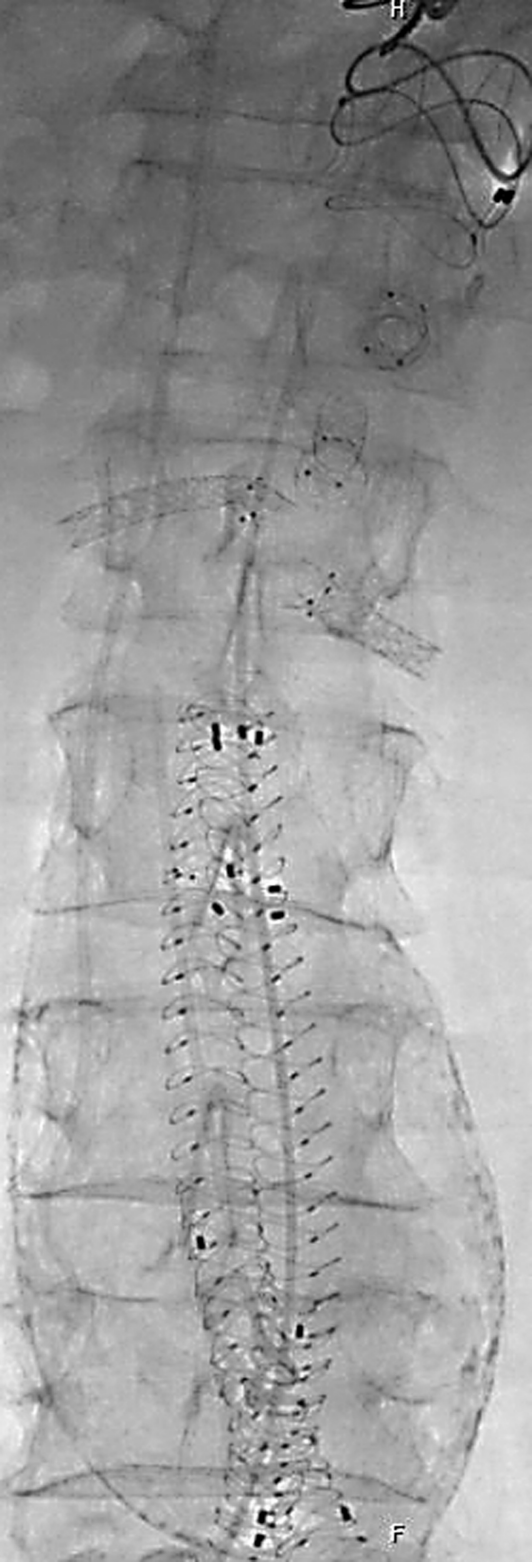
Anterior–posterior fluoroscopy of the abdomen showing the final result after implantation of the fenestrated Anaconda with four bridging stent grafts: celiac trunk—BF 8/10 × 27 mm, superior mesenteric artery BF 7/10 × 27 mm, right renal artery—BGP 5 × 38 mm, left renal artery—BGP 6 × 28 mm. BF, BeFlared.

Three months after the patient's initial presentation, the last operation was performed. First, the DFA aneurysm was repaired with a 7-mm Polytetrafluoroethylene (PTFE) profundo-profundal interposition graft. Because of the high femoral bifurcation and good control due to the open surgical exposition as well as sufficient vessel diameter, the proximal superficial femoral artery (SFA) was used as the access vessel for FEVAR on the left. On the right side, which was the main device introduction site, a percutaneous approach with ultrasound-guided puncture of the Dacron graft was used. After predilatation with 11F, 16F, and 18F sheaths, the 22F main device was introduced without problems. The puncture site was later closed surgically. According to our routine protocol for FEVAR, the patient had a prophylactic spinal fluid drain placed 1 day preoperatively. The operation was performed with intraoperative monitoring of spinal cord function using motor-evoked potentials (MEPs; iNCU GmbH, Emmendingen, Germany) and distal perfusion of the legs via antegrade sheaths (6F and 7F) placed in the SFA. All target vessels were easily cannulated from the femoral access using a 7.5 F steerable sheath (Oscor Inc., Palm Harbor, FL). As target vessel stent grafts, we used the novel BF stent graft (Bentley InnoMed, Hechingen, Germany) for the celiac trunc (CT: 8/10 × 27 mm) and superior mesenteric artery (SMA: 7/10 × 27 mm), and BeGraft Peripheral (BGP) stent grafts (Bentley InnoMed, Hechingen, Germany) for the renal arteries (RAs, right: 5 × 38 mm, flared to 8 mm; left: 6 × 28 mm, flared to 8 mm). Routinely, before stenting the last fenestration, we complete the FEVAR bifurcation so that we can then evaluate the MEPs during a 15-minute test occlusion. After performing this step, a 50% MEP drop was noted only in the right leg, which did not change during the test occlusion. It was interpreted as moderate peripheral leg ischemia due to the large sheath in the CFA in combination with the known PAA occlusion on that side and despite the distal femoral perfusion. Thus, the FEVAR procedure was completed by stenting the right RA ( Fig. 2 ). The completion angiogram showed a good result with only a slight type II endoleak. The total operating time was 356 minutes, mainly due to the lengthy reconstruction of the left DFA in the beginning. Total fluoroscopy time, radiation dose, and amount of contrast agent were 39 minutes, 9,209.74 μGray*m ^2^ , and 90 mL, respectively.

The patient's early postoperative course was uneventful, especially without any kind of ischemic symptoms (spinal cord or arterial).

## Discussion

This case, demonstrating the successful staged treatment of an aged patient with polyaneurysmal disease, is of high educational value because it assembles multiple vascular pathologies and types of surgical treatment. Further, it is unique for reporting the world's first implantation of the novel BF stent graft (Bentley InnoMed, Hechingen, Germany) as a bridging stent graft in an Anaconda FEVAR. Both aspects will be discussed in the following paragraphs.

PAAs are known for their embolization risk and a primary presentation with claudication symptoms, as well as bilateral presence and association with AAA, as in this case, are common. 4 Surgical treatment is recommended in PAA >20 mm, all symptomatic cases, and cases with the presence of thrombus to prevent embolic complications. Performing an interposition graft, with autologous vein as the best graft material in terms of long-term patency, combined with proximal and distal aneurysm ligation, is the standard of care, and both medial and dorsal approaches are possible. The dorsal approach that allows for complete ligation of side branches may be advantageous in the long run and is easily performed in PAA of limited longitudinal extent, as in the present case. 5

In addition to bilateral PAA and a juxtarenal AAA, the present patient had an iliac, a CFA, and a DFA aneurysm. This polyaneurysmal disease is typically found in patients of higher age with arterial hypertension and a family history of cardiovascular or aneurysm disease. 3 A genetic disposition is rarely found or tested for in those patients 3 6 and was not performed either in the present case. The indication for surgical repair of the CFA and DFA aneurysms in this case was given due to the aneurysm size and associated risk of aneurysm growth and rupture, especially for the DFA aneurysm, 2 as well as due to the claudication symptoms and postaneurysm stenosis and in order to prepare the access site for FEVAR on the right side. It may be discussed whether separating the DFA aneurysm repair and FEVAR procedure, so as to reduce the individual operating time, would have been advantageous. But then, the time until exclusion of the large juxtarenal AAA would have been prolonged additionally, increasing the risk of rupture in the interim. We aimed at minimizing this time by timing the operative steps adequately with the planning and ordering of the custom-made FEVAR graft. Open surgery for the juxtarenal AAA, obviously having the shortest waiting time, was deemed inadequate in this case due to the patient's age and cardiac history. However, all therapeutic options and their respective advantages and risks were presented to the patient preoperatively and discussed in detail, leading to the herein presented treatment plan.

While discussing all technical aspects of the FEVAR procedure in this report would be far too extensive, the most important and unique aspect of the present case, namely the world's first use of the novel BF bridging stent graft, has to be highlighted.

The introduction of covered bridging stent grafts for the visceral target vessels in 2004 has revolutionized FEVAR, and devices from several manufacturers are now available. Recently, the BGP stent graft was the first to obtain conformité européenne (CE) marking for use as a bridging stent graft in FEVAR. In order to achieve optimal sealing at the level of the fenestrations, the stent grafts need to be flared with a larger balloon, typically 2 mm larger in diameter. For several years now, the development of flared bridging stent grafts has been ongoing so as to eliminate the necessity for balloon exchange, thereby facilitating the already complex FEVAR procedure. Since this very month, the BF is the first such stent graft on the market, and the first in human implantation was performed on November 25, 2024, in a Cook (Cook Australia Pty Ltd, Brisbane, Australia) FEVAR with success.

**Fig. 3:**
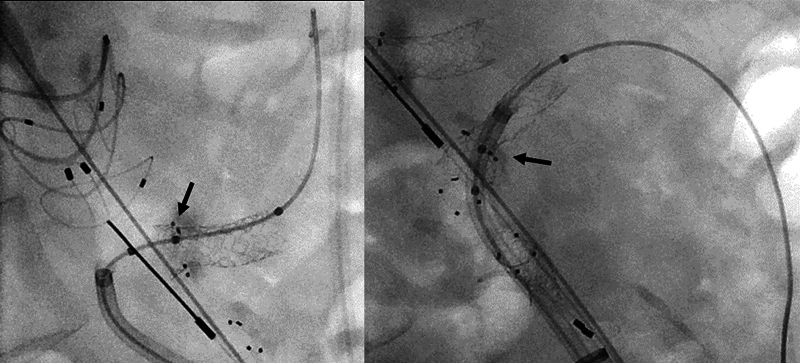
Sagittal fluoroscopy showing the implanted BF stent grafts in the celiac trunk (left) and superior mesenteric artery. The stent grafts have been positioned precisely with the middle marker in-line with the fenestration (arrow). BF, BeFlared.

Due to the flared design, this stent graft has to be placed very precisely with the respective marker at the level of the fenestration ( Fig. 3 ). Since the Anaconda FEVAR has an unsupported main body in contrast to the Cook device, this might be more challenging in theory. In this first implantation, the precise positioning and deployment was however without any problems and showed an excellent intraoperative result ( Fig. 3 ). The smaller BF sizes not being available yet, the renal fenestrations in the present case were stented using BGP stent grafts with subsequent flaring in the traditional manner and the direct intraoperative comparison made the practicality of the BF evident. Although the effect on total procedure and radiation time was not apparent in the present case due to other reasons, it is to be expected.

In conclusion, this case demonstrates how presumably simple symptoms may turn out to be a complex problem. However, thorough case planning and the combination of different vascular surgical techniques and devices, from traditional to brand new, allow for successful therapy also of an aged patient. The continued development of technologies and devices is expected to facilitate complex procedures like FEVAR further in the future.
